# A Quantum Predictive Brain: Complementarity Between Top-Down Predictions and Bottom-Up Evidence

**DOI:** 10.3389/fpsyg.2022.869894

**Published:** 2022-07-08

**Authors:** Antonio Mastrogiorgio

**Affiliations:** IMT School for Advanced Studies Lucca, Lucca, Italy

**Keywords:** predictive brain, neural reuse, Quantum Predictive Brain, complementarity, quantum cognition

## Abstract

Predictive brain theory challenges the general assumption of a brain extracting knowledge from sensations and considers the brain as an organ of inference, actively constructing explanations about reality beyond its sensory evidence. Predictive brain has been formalized through Bayesian updating, where top-down predictions are compared with bottom-up evidence. In this article, we propose a different approach to predictive brain based on quantum probability—we call it Quantum Predictive Brain (QPB). QPB is consistent with the Bayesian framework, but considers it as a special case. The tenet of QPB is that top-down predictions and bottom-up evidence are complementary, as they cannot be co-jointly determined to pursue a univocal model of brain functioning. QPB can account for several high-order cognitive phenomena (which are problematic in current predictive brain theories) and offers new insights into the mechanisms of neural reuse.

*“It’s not the deer that crosses the road, it’s the road that crosses the forest”[unknown author]*

## Introduction

Although we know that the sun is fixed and the horizon moves, we observe that the sun goes down behind a stationary horizon. More than 150 years have passed since the German physicist Hermann von Helmholtz described the involuntary mechanisms involved in visual impressions (see Dayan et al., 1995). Being humans, we are often involved in situations (for instance, optical illusions) in which the sensory evidence, which follows its own strict rules, seems to outclass our rational expectations. The flourishing theory of predictive brain (e.g., Friston, 2009; Clark, 2013)—a brain constantly matching top-down expectations with bottom-up sensory inputs—is sympathetic to Helmholtz’s ideas and represents a powerful theoretical device, able to challenge the traditional principles of a brain building models of the world by accumulating cues through bottom-up processes. According to this view, the brain is a predictive machine reactive to “surprises” and works through a hierarchical generative model aiming to minimize prediction error. Any adaptive change minimizes free-energy, that is to say, the brain works through conservative principles (Friston, 2012, 2009).

But, involuntary mechanisms are not the end of the story. What about higher-order illusions? Let us consider the incipit of this contribution “It’s not the deer that crosses the road, it’s the road that crosses the forest.” It summons up, by means of a high-level “illusion,” the loss of awareness of the ecological embeddedness of human beings. This kind of illusion is quite different to Helmholtz’s one about the sun and the horizon. There is no sensory illusion, as the deer actually crosses the road. But, the illusion starts to be there exactly when one oversteps the veil of sensory obviousness. Indeed, the point is not just a mere spatial recognition of “who (unexpectedly) moves with respect to what,” but that this recognition is somehow evocative and poetically meaningful.

The theory of predictive brain is still in its infancy for such kinds of phenomena involving higher-order processes and complex sense-making. A deer crossing the road is, for sure, unexpected, but this is relatively trivial. The most interesting surprise lies in the higher-order discovery of the ecological embeddedness, occurring when one realizes that the road is nothing more than a small line in a huge forest. A deer crossing the road is, to a different cognitive order, illusory once one realizes that the driver’s point of view is *incompatible* with respect to the forest’s (and to the deer’s) point of view. Put in different words, we can hypothesize that the driver’s surprise lies in experiencing two incompatible points of view—the driver’s and the forest’s—and they are salient exactly because they cannot be reduced to a unique model.

The Bayesian framework in predictive brain theory represents a significant methodological device to account for surprise—technically speaking “surprisal” (Clark, 2013)—as the “unexpected” is used to update the model. However, a Bayesian framework is somehow inadequate to model such kinds of phenomena involving unconventional surprises, incommensurable points of view and sophisticated contemplative experience, which are a mark of the human being. In this contribution, we propose a novel approach to predictive brain—we call it Quantum Predictive Brain (QPB)—that exploits the formalism of quantum cognition (Busemeyer and Bruza, 2012). The fundamental idea of QPB is that *top-down predictions and bottom-up evidence are complementary*; we can determine the state of the top-down system only if we accept some non-reducible uncertainty about the state of the bottom-up system, and vice versa (for complementarity, see Plotnitsky, 2014; Wang and Busemeyer, 2015). Far from being just a speculative exploration (based on the use of a non-Kolmogorovian, quantum probability), as we will explain in the next sections, the theoretical reasons for a quantum approach to predictive brain are both neurocognitive and psychological.

### Predictive Coding

Predictive brain represents an alternative to the orthodox framework based on the general assumption that sensory inputs “ascend” by integrating the complexity of perceptual experience. Predictive brain proposes an inverse explanation as “the brain became an organ of inference, actively constructing explanations for what’s going on ‘out there’, beyond its sensory epithelia” (Friston, 2018, p. 1019). Predictive brain challenges the general assumption that the brain is a device that extracts knowledge from sensations, and proposes a unifying theory of cortical function postulating that the core function of the brain is the *minimization of the prediction error*, which represents the mismatch between the predicted input and the actual evidence (Rao and Ballard, 1999; Friston, 2009; Clark, 2013). In this view, the brain is composed of a hierarchy of layers, where each layer makes predictions about the layers below in the hierarchy. A prediction error—the information that, at each layer, is not successfully predicted—is formed through a comparison between the downward descending predictions and the activity of the layer. Prediction errors serve as input to higher levels and can be used to update the mental model of the world. Generally speaking, by minimizing the prediction error, the brain performs perceptual inference and learning, as predictions are instantiated at multiple scales and hierarchy levels, from the low-level variation of sensory data to high-level models of causes of sensory data (Clark, 2015). Prediction error minimization is affected by *precision*, related to the “signal to noise ratio” in updating mechanisms. Attention can be considered as the inference of precision in hierarchical perception (Feldman and Friston, 2010; see also Hohwy, 2012; Yon and Frith, 2021). In more detail, prediction error minimization passes through the inference of hidden external states (perception) through updating the model of the world (learning) or through the actions employed to conform to predictions. Despite the elegance of the predictive brain framework, its neurobiological dimension is nowadays subject to increasing investigation, as the evidence is still mixed (Walsh et al., 2020).

The principles of predictive brain present important theoretical antecedents—in particular, Kant’s *a priori* knowledge, Helmholtz’s unconscious inference, and the notion of feedback control in cybernetics. The modern mathematical formalization of predictive brain—also known as predictive coding (Huang and Rao, 2011)—is relatively recent. After the seminal article of Rao and Ballard (1999), such formalization passed through the recognition that predictive coding could be approximated by Bayesian inference based upon Gaussian generative models (e.g., Friston, 2003, 2008). As discussed by Friston (2012, p. 1230): “Bayesian brain is a corollary of the free energy principle, which says that any self organizing system (like a brain or neuroimaging community) must maximize the evidence for its own existence, which means it must minimize its free energy using a model of its world.” The current mathematical formalization of predictive brain is not secondary to its theoretical tenets and its well-developed formalism often represents a barrier for the outsiders. Maximizing Bayesian evidence (or minimizing variational free energy) has been described as a gradient descent minimization of variational free energy (Friston et al., 2017), able to account for several phenomena such as “repetition suppression, omission responses, violation responses, place cell activity, phase precession, theta sequences, theta-gamma coupling, evidence accumulation, race-to-bound dynamics, and transfer of dopamine responses” (Friston et al., 2017, p. 37). Generally speaking, the minimization of variational free energy allows a parsimonious but accurate description of observable outcomes of different types.

Bayesian inference does not necessarily need to be implemented by predictive coding (Aitchison and Lengyel, 2017). Millidge et al. (2021) propose a review of the mathematical components of predictive coding. Predictive coding can be considered, in its foundational principle, as a form of variational inference (under Gaussian assumptions) where multi-layered topology, dynamical properties, the multiplicity of stimulus, and precision are essential factors. Interestingly, Millidge et al. (2021) discuss the future directions of research on predictive coding, identifying the following domains: the plausible existence of non-Gaussian but discrete generative models at the highest cognitive levels, the necessity of formalizing the basis of long term memory involved in cortical working, the opportunity of considering the actual architecture of the cortex (which could not be fully connected through hierarchical layers, but presents sparse connectivity and “columnar” structures), and the implementation of precision consistent with known neurobiological mechanisms. Furthermore, they identify several machine learning algorithms that could be comparatively used to shed light on updating mechanisms involved in predictive coding.

Current literature on predictive coding (extensively discussed by Millidge et al., 2021) never raises the more foundational issues about the very nature of probability, assumed in predictive coding. In particular, current research in predictive brain does not consider the existence of alternative probabilistic frameworks, which are not based on Kolmogorov’s probability axioms and Boolean logic, but informed by quantum principles. Among the exceptions, Fields et al. (2021) discuss quantum systems as observers assigning semantics to observational outcomes; the authors emphasize that context-switching challenges the classical formulation of the free-energy principle so as to justify a quantum formulation. Such a quantum approach represents a novel and promising perspective for future research on the free energy principle. Generally speaking, quantum mechanics can be used to solve the so-called “Bayesian blur” problem—related to the contrast between the univocal nature and the probabilistic roots of human experience (Clark, 2018)—where consciousness is considered a “collapsing agent” (Safron, 2021), which is consistent with von Neumann & Wigner’s interpretation, according to which, consciousness is necessary for quantum measurement (von Neumann, 1932/1955; Wigner and Margenau, 1967).

The QPB—presented in the next section through a stylized model—aims to update the current view of the predictive brain through a quantum probabilistic framework.

## Quantum Predictive Brain: A Stylized Model

The standard formalization of probability—in which the Bayesian framework is conceived—relies on Kolmogorov’s probability axioms. But, Kolmogorov axioms are not the unique way to formalize probability. Quantum theory represents a significant alternative (relatively under-explored in disciplines other than physics) with interesting applications in the cognitive realm, also known as quantum cognition (Aerts, 2009; Busemeyer and Bruza, 2012; Khrennikov, 2015; cf. Atmanspacher et al., 2002). Also, quantum cognition proposes a different cognitive explanation to many factual phenomena—mainly related to judgments under uncertainty (Kahneman et al., 1982)—traditionally conceived in a Bayesian framework (for a comparison between Bayesian and quantum frameworks, see Bruza et al., 2015). Generally speaking, quantum measurement can be conceived as Bayesian updating where “collapse”—to be generally intended as the transition from a “superposition,” indefinite state to a definite state associated with the observed outcome—corresponds to a revision of the state conditioned to new information (Caves et al., 2002; Busemeyer and Bruza, 2012).

In the next sections, we present a stylized model of QPB along with a gradual introduction of the fundamental concepts of quantum cognition, formalized through a geometric approach. Such a geometric approach has been readapted from Busemeyer and Bruza (2012, Section 2.1), which is an excellent contribution to exploring the potential of quantum models in cognitive science (for a geometric approach, see also Franco, 2009; Lambert-Mogiliansky et al., 2009). While classical probability is formalized using events (employing set theory), quantum formalism uses spaces and projections on subspaces and presents a different geometric interpretation.

Quantum formalism is supposed to be non-friendly and almost inaccessible as it requires dedicated mathematical tools. Actually, it constitutes a well-established domain of knowledge. von Neumann (1932/1955) provided the mathematical formalism of quantum theory. Linear algebra represents a suitable tool to model quantum phenomena. Among the various notations used to express quantum concepts, we will refer to Dirac’s formalism (Dirac, 1939), also known as *bra-ket notation*.

### Top-Down and Bottom-Up Systems

In each instant, an organism is in a state |*S*⟩, which is a unit-length vector, expressed in bra-ket notation, in an *N*-dimensional Hilbert space, representing the states of the system. A Hilbert space is a generalization of Euclidean space and was originally used to formalize quantum mechanics (for a detailed formulation of the nature of events and their properties in a Hilbert space, see Busemeyer and Bruza, 2012, Sections 2.1 and 2.5). The *N* dimensions of this space represent potential states that are possible for an organism and the unit-length vector state represents the state of the system. In our model, we do not consider that the dimensions could be infinite and include real or complex coefficients; neither do we specify additional constraints. This deliberate simplification is consistent with the geometric approach (proposed by Busemeyer and Bruza, 2012, Section 2.1) and is suitable to introduce quantum cognition to non-experts (as we assume that predictive coding scholars are not versed in quantum formalism).

The dimensions of the system must be conceptualized as the possible values of the neurobiological degrees of freedom, where events can be of two general types: top-down predictions or bottom-up evidence. As we explain in the following sections, the fundamental idea of a quantum view of predictive brain is that this state admits incompatible—*complementary*—representations depending on the fact that we are considering top-down or bottom-up processes.

#### Top-Down System

Let us consider the system with the potential predictions *P1* and *P2*. Notice that, for simplicity, we are choosing two options, but this logic applies to a space with an arbitrary number of dimensions and can be generalized to arbitrary numbers of predictions *P1*, *P2*, *P3*, etc. A generalization to *N* dimensions should be consistent with neuroscientific evidence and is not part of the present model.

**FIGURE 1:**
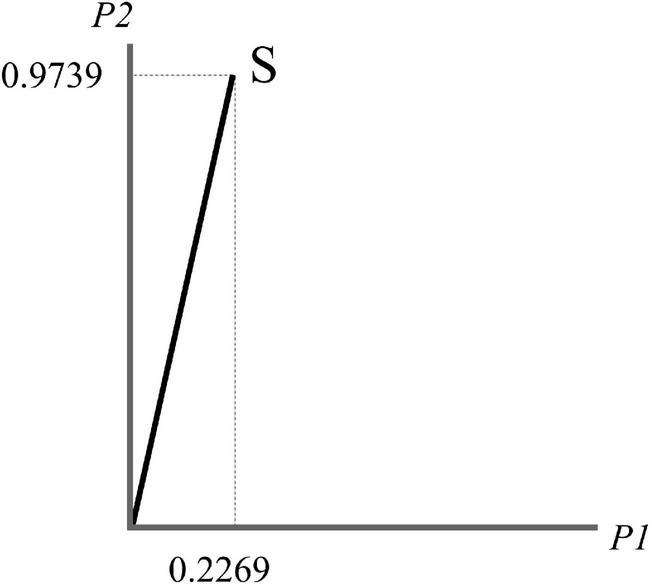
State of the top-down system with predictions (P).

The two vectors {|*P*1⟩,|*P*2⟩} are a *basis*. So, an arbitrary vector in their space can be expressed by a linear combination of them. *S*, the unit-length vector, representing the state of the system, is a combination of {|*P*1⟩,|*P*2⟩} such as:


(1) |S⟩=(0.2269)⋅|P⁢1⟩+(0.9739)⋅|P⁢2⟩

where


(2) $$\psi =\left[\begin{array}{c} \psi_{1}\\ \psi_{2} \end{array}\right]=\left[\begin{array}{c} 0.2269\\ 0.9739 \end{array}\right]$$

represents the coordinates of the state |*S*⟩ with respect to {|*P*1⟩,|*P*2⟩}, as in Figure 1.

Quantum theory works differently from classical probability. *S* is in an indefinite state, formally called a *superposition* state, before the collapse—to generally indicate any state that eventuates, after some processing, in a definite output—occurs. In other words, an indefinite state allows all the definite states to have the potential for being expressed at each moment, as superposition expresses the psychological state of conflict and ambiguity between potential observable states.

The squared length of the unit-length vector *S*, representing the state of the system, equals 1 by definition, being the sum of the squared magnitudes of its coordinates:


(3) $${||\left|S\right\rangle ||}^{2}={||\psi ||}^{2}=\left({\left|0.2269\right|}^{2}+{\left|0.9739\right|}^{2}\right)=1$$

According to quantum formalism—specifically the Born rule, Born (1926)—if we project the state on a subspace and then we square, the result is the probability of its occurrence. Hence, the probability of the prediction *P1* is obtained by considering the projection of *S* on the subspace *P1* to identify, on the *P1-*axis, the relative segment. If we square the length of the projection, we obtain the probability that the specific prediction *P1* occurs. Notice that we could express (one or more) coordinates also in negative values (for instance 0.2269, −0.9739) and we should draw the respective negative axis as in Figure 1. This modification is irrelevant because inverting the direction of one or more vectors, for example from |*P*2⟩ to −|*P*2⟩, does not affect the probability (since it is the square of the amplitude); the new basis is “equivalent” to the first one as both vectors span the same ray. In our model, *S* can be expressed equivalently both in the {|*P*1⟩,|*P*2⟩} and {|*P*1⟩,−|*P*2⟩} basis, or in any other basis obtained by this type of transformation.

Formally speaking, we know that with respect to the {|*P*1⟩,|*P*2⟩} basis, the coordinates of each vector are represented by a canonical system:


(4) $$\left|P\,1\right\rangle \to \left[\begin{array}{c} 1\\ 0 \end{array}\right],\left|P\,2\right\rangle \to \left[\begin{array}{c} 0\\ 1 \end{array}\right]$$

The inner product ⟨*S*|*X*⟩, called *transition amplitude*, represents the amplitude from |*S*⟩ to |*P*1⟩:


(5) $$\left\langle S|P\,1\right\rangle =\left[\begin{array}{cc} 10 & \end{array}\right]\cdot \left[\begin{array}{c} 0.2269\\ 0.9739 \end{array}\right]==\left(1\cdot 0.2269\right)+\left(0\cdot 0.9739\right)==0.2269$$

If we project |*S*⟩on the *p1*-ray, we obtain:


(6) |P⁢1⟩⁢⟨S|P⁢1⟩=0.2269⋅|P⁢1⟩

and its squared length is:


(7) $${||0.2269\cdot \left|P\,1\right\rangle ||}^{2}=0.0515$$

which represents the probability of *P1*.

As the two predictions *P1* and *P2* orthogonal, in quantum formalism, the probability of both is simply related to the sum of both events:


(8) $${||\left(0.2269\right)\cdot \left|P\,1\right\rangle +\left(0.9739\right)\cdot \left|P\,2\right\rangle ||}^{2}=0.0515+0.9485=1$$

Notice that we can also compute the probability for more general events represented by subspaces in an *N*-dimensional space. For instance, in a 5-dimensional space, the projection of the union “*P1* or *P2* or *P3*” is (|*P*1⟩⟨*P*1| + |*P*2⟩⟨*P*2| + |*P*3⟩⟨*P*3|)|*S*⟩=|*P*1⟩⟨*P*1|*S*⟩ + |*P*2⟩⟨*P*2|*S*⟩ + |*P*3⟩⟨*P*3|*S*⟩=(ψ_1_)⋅|*P*1⟩ + (ψ_2_)⋅|*P*2⟩ + (ψ_3_)⋅|*P*3⟩, and the probability is ||(ψ_1_)⋅|*P*1⟩ + (ψ_2_)⋅|*P*2⟩ + (ψ_3_)⋅|*P*3⟩||2 (as orthogonality guarantees that the squared length of the sum equals the sum of squared lengths).

#### Bottom-Up System

Now let us consider the evidence *E1* and *E2*. As in the case of top-down processes, this formalization can be generalized to arbitrary numbers of evidence, *E1*, *E2*, *E3*, etc.

**FIGURE 2:**
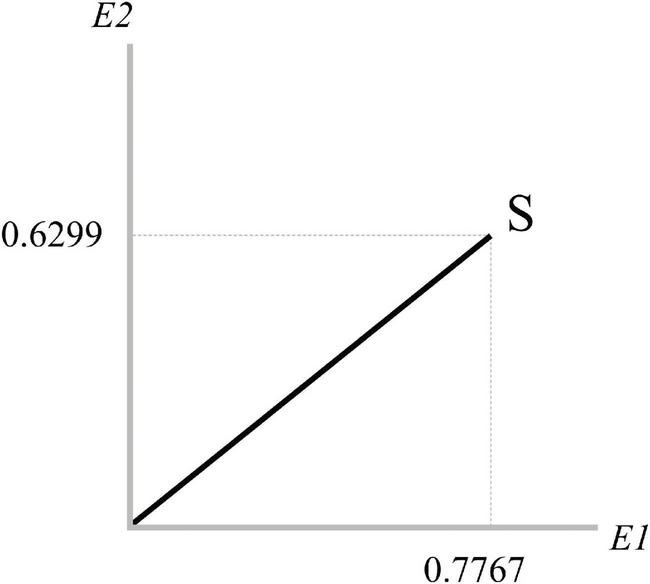
State of the bottom-up system with evidence (E).

The two vectors {|*E*1⟩,|*E*2⟩} are the basis of the bottom-up system and an arbitrary vector in their space can be expressed by a linear combination of them. Notice that *S*, the unit-length vector, representing the state of the system in the previous top-down system, is expressed in the bottom-up system as a combination of {|*E*1⟩,|*E*2⟩} such as:


(9) |S⟩=(0.7767)⋅|E⁢1⟩+(0.6299)⋅|E⁢2⟩

where


(10) $$\varphi =\left[\begin{array}{c} \varphi_{1}\\ \varphi_{2} \end{array}\right]=\left[\begin{array}{c} 0.7767\\ 0.6299 \end{array}\right]$$

represents the coordinates of the state |*S*⟩ with respect to {|*E*1⟩,|*E*2⟩}, as in Figure 2.

As in the case of prediction (discussed in the previous section), here, *S* is in a superposed state as evidence could be potentially expressed through the collapse.

The squared length of the unit-length vector *S*, representing the state of the system, equals 1 by definition, being the sum of the squared magnitudes of its coordinates:


(11) $${||\left|S\right\rangle ||}^{2}={||\varphi ||}^{2}=\left({\left|0.7767\right|}^{2}+{\left|0.6299\right|}^{2}\right)=1$$

Again, if we project the state *S* on a subspace and then we square, the result is the probability of its occurrence. Concerning the {|*E*1⟩,|*E*2⟩} basis, the coordinates of each vector are represented by a canonical system:


(12) $$\left|E\,1\right\rangle \to \left[\begin{array}{c} 1\\ 0 \end{array}\right],\left|E\,2\right\rangle \to \left[\begin{array}{c} 0\\ 1 \end{array}\right]$$

The inner product ⟨*S*|*E*1⟩ represents the amplitude from |*S*⟩ to |*E*1⟩:


(13) $$\left\langle S|E\,1\right\rangle =\left[\begin{array}{cc} 10 & \end{array}\right]\cdot \left[\begin{array}{c} 0.7767\\ 0.6299 \end{array}\right]==\left(1\cdot 0.7767\right)+\left(0\cdot 0.6299\right)==0.7766$$

If we project |*S*⟩on the *E1*-ray we obtain:


(14) |E⁢1⟩⁢⟨S|E⁢1⟩=0.7767⋅|E⁢1⟩

and its squared length is:


(15) $${||0.7767\cdot \left|E\,1\right\rangle ||}^{2}=0.6032$$

which represents the probability of *E1*.

As the two evidence *E1* and *E2* are orthogonal, in quantum formalism, the probability of both is simply the sum of both events:


(16) $${||\left(0.7767\right)\cdot \left|E\,1\right\rangle +\left(0.6299\right)\cdot \left|E\,2\right\rangle ||}^{2}=0.6032+0.3968=1$$

The union of the events is the sum of the probabilities related to its orthogonal sub-dimensions.

Notice that, as in the case of top-down processes, we can also compute the probability for more general events represented by subspaces in an *N*-dimensional space. For instance, in a 5-dimensional space, the projection of the union “*E1* or *E2* or *E3*” is (|*E*1⟩⟨*E*1| + |*E*2⟩⟨*E*2| + |*E*3⟩⟨*E*3|)|*S*⟩=|*E*1⟩⟨*E*1|*S*⟩ + |*E*2⟩⟨*E*2|*S*⟩ + |*E*3⟩⟨*E*3|*S*⟩=(φ_1_)⋅|*E*1⟩ + (φ_2_)⋅|*E*2⟩ + (φ_3_)⋅|*E*3⟩, and the probability is ||(φ_1_)⋅|*E*1⟩ + (φ_2_)⋅|*E*2⟩ + (φ_3_)⋅|*E*3⟩||2 (as orthogonality guarantees that the squared length of the sum equals the sum of squared lengths).

### Complementarity Between Top-Down and Bottom-Up Systems

Predictive coding is based on the hypothesis that the brain minimizes the prediction errors with respect to a generative model of the world. Both classical and quantum theories provide a tool for model updating the state conditioned to some observations. In a classical Bayesian framework, when an event is observed, the original probability function is changed into a new conditional function. In particular, the joint probability is normalized and this guarantees that the new probability function sums to one. Quantum theory proposes, *mutatis mutandis*, a similar model: the original state vector is transformed into a new state vector through a projection. In particular, the original state is projected onto the subspace of the observed event and then such projection is divided by its length. This transformation of the state vector onto a subspace corresponding to the observed event is called “projective measurement.” In our stylized model, we call it *collapse* to generally indicate any state that eventuates, after some processing, in a definite output. In the words of Busemeyer and Bruza (2012, p. 23), collapse refers to “the transition from a superposition state to a definite state associated with the observed outcome.”

The difference between a classical and a quantum framework is marginal when the systems are compatible (the probability of the union of independent events is simply their sum, and the probabilities of all the events sum to unity). However, things change when we consider complementary systems, that is to say, predictions and evidence are *incompatible*. The certainty about predictions may lead to uncertainty about evidence (and vice versa), as incompatibility is, in the words of Busemeyer and Bruza (2012, p. 23), “mathematically implemented by the non-commutativity of quantum measurements.” Indeed in case of incompatibility, predictions and evidence do not share the same basis, but require separate spaces. In particular, each one (predictions and evidence) considered separately admits a Boolean framework, but they cannot be treated in a Boolean way once they are “pasted together.” A quantum probabilistic approach presents more flexibility than the Bayesian, as it does not require specifying the joint probabilities. More specifically, as we explain in section “The Role of Quantum Contextuality”, the notion of “quantum contextuality”—whose role is nowadays debated with reference to quantum evidence in the behavioral domain (e.g., Dzhafarov et al., 2016)—represents a fundamental theoretical device able to provide alternative explanations to incompatibility.

Incompatibility is central in the QPB and represents a substantial innovation compared to existing theories of Bayesian predictive coding, assuming a unique space for both predictions and evidence. We know from the previous two sections that *S*—the vector representing the state of the system—can be represented in terms of either the top-down system (that is {|*P*1⟩,|*P*2⟩} basis) or the bottom-up system (that is {|*E*1⟩,|*E*2⟩} basis). Indeed, we know that:


(17) |S⟩=(0.2269)⋅|P⁢1⟩+(0.9739)⋅|P⁢2⟩

and


(18) |S⟩=(0.7767)⋅|E⁢1⟩+(0.6299)⋅|E⁢2⟩

so


**FIGURE 3:**
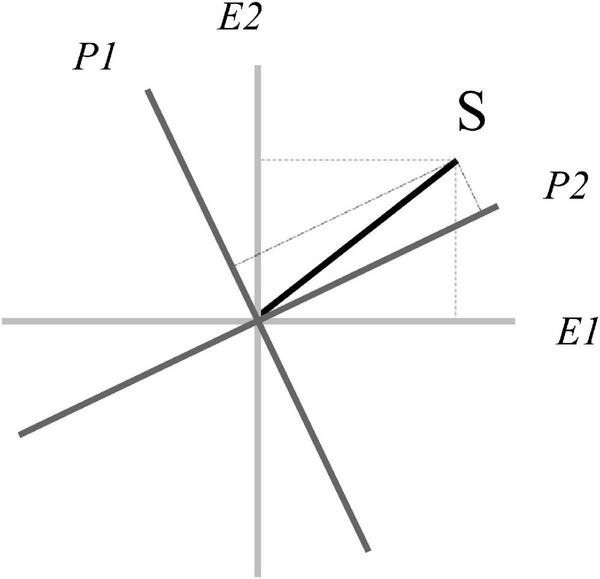
Incompatibility between predictions (P) and evidence (E).

(19) |S⟩=(0.2269)⋅|P⁢1⟩+(0.9739)⋅|P⁢2⟩=(0.7767)⋅|E⁢1⟩+(0.6299)⋅|E⁢2⟩

where


(20) $$\psi =\left[\begin{array}{c} \psi_{1}\\ \psi_{2} \end{array}\right]=\left[\begin{array}{c} 0.2269\\ 0.9739 \end{array}\right]$$

represents the coordinates of the state |*S*⟩ with respect to {|*P*1⟩,|*P*2⟩}, and


(21) $$\varphi =\left[\begin{array}{c} \varphi_{1}\\ \varphi_{2} \end{array}\right]=\left[\begin{array}{c} 0.7767\\ 0.6299 \end{array}\right]$$

represents the coordinates of the state |*S*⟩ with respect to {|*E*1⟩,|*E*2⟩}. Now we know that the same state |*S*⟩ can be represented using two incompatible bases, that is to say, we have different coordinates depending on the basis, as in Figure 3.

We can represent the basis vectors of the top-down system in terms of the bottom-up system basis:


(22) |P⁢1⟩=(-0.4372)⋅|E⁢1⟩+(0.8994)⋅|E⁢2⟩

(23) |P⁢2⟩=(0.8994)⋅|E⁢1⟩+(0.4372)⋅|E⁢2⟩

And, vice versa, we can represent the basis vectors of the bottom-up system in terms of the top-down system basis:


(24) |E⁢1⟩=(-0.4372)⋅|P⁢1⟩⁢(0.8994)⋅|P⁢2⟩

(25) |E⁢2⟩=(0.8994)⋅|P⁢1⟩+(0.4372)⋅|P⁢2⟩

We can express each system with respect to the other. If we want to express the coordinates of the bottom-up system with {|*E*1⟩,|*E*2⟩} basis in terms of the top-down system with {|*P*1⟩,|*P*2⟩} basis, we have:


(26) $$\psi =U_{P\,E}\cdot \varphi \left[\begin{array}{c} \psi_{1}\\ \psi_{2} \end{array}\right]=\left[\begin{array}{cc} \left\langle P\,1|E\,1\right\rangle \,\left\langle P\,1|E\,2\right\rangle & \\ \left\langle P\,2|E\,1\right\rangle \,\left\langle P\,2|E\,2\right\rangle & \end{array}\right]\cdot \left[\begin{array}{c} \varphi_{1}\\ \varphi_{2} \end{array}\right]$$

And if we want to express the coordinates of the top-down system with {|*P*1⟩,|*P*2⟩} basis in terms of bottom-up system with {|*E*1⟩,|*E*2⟩} basis, we have:


(27) $$\varphi =U_{P\,E}^{\text{†}}\cdot \psi \left[\begin{array}{c} \varphi_{1}\\ \varphi_{2} \end{array}\right]=\left[\begin{array}{cc} \left\langle E\,1|P\,1\right\rangle \,\left\langle E\,1|P\,2\right\rangle & \\ \left\langle E\,2|P\,1\right\rangle \,\left\langle E\,2|P\,2\right\rangle & \end{array}\right]\cdot \left[\begin{array}{c} \psi_{1}\\ \psi_{2} \end{array}\right]$$

where † indicates the transpose-conjugate operation, as $U_{P\,E}\cdot U_{P\,E}^{\text{†}}=I$, where *I* is an identity matrix.

The incompatibility between the prediction system and evidence system, in Figure 3, represents a form of fundamental uncertainty, related to the famous Heisenberg uncertainty principle. Indeed, if we are certain in the evidence system—we observe a specific sensory input—we must accept some uncertainty in the prediction system—different predictions are still potential—and vice versa. In other words, once the state vector collapses in a system, it remains in a superposed state in the complementary system. This point is a tenet of the QPB, positing that we can determine the state of the top-down system only if we remain uncertain about the state of the bottom-up system, and vice versa.

#### Order Effects and Interference

A fundamental implication of complementarity lies in the presence of non-commutativity between events (explained in section “Complementarity Between Top-Down and Bottom-Up Systems”), which gives rise to *order effects* (discussed in Wang and Busemeyer, 2013). Let us consider the state from the top-down system. Firstly, we want to analyze the transition from *S* to *P1* passing through the state E2:


(28) |S⟩→|E⁢2⟩→|P⁢1⟩

In this case, we first have to project the *S* state on the *E2* ray:


(29) $$\left\langle E\,2|S\right\rangle =\left[\begin{array}{cc} 0.89940.4372 & \end{array}\right]\,\left[\begin{array}{c} 0.2269\\ 0.9739 \end{array}\right]=\left(0.8994\right)\cdot \left(0.2269\right)+\left(0.4372\right)\cdot \left(0.9739\right)=0.6299$$

The probability, as explained above, is:


(30) $${\left|\left\langle E\,2|S\right\rangle \right|}^{2}={\left|0.6299\right|}^{2}=0.3968$$

Now we have to analyze the path E2 to P1:


(31) $$\left\langle P\,1|E\,2\right\rangle =\left[\begin{array}{cc} 0.89940.4372 & \end{array}\right]\,\left[\begin{array}{c} 1\\ 0 \end{array}\right]=\left(0.8994\right)\cdot \left(1\right)+\left(0.4372\right)\cdot \left(0\right)=0.8994$$

And the probability is:


(32) $${\left|\left\langle P\,1|E\,2\right\rangle \right|}^{2}={\left|0.8994\right|}^{2}=0.8089$$

Hence the probability of the path|*S*⟩→|*E*2⟩→|*P*1⟩ is:


(33) $${\left|\left\langle P\,1|E\,2\right\rangle \right|}^{2}\cdot {\left|\left\langle E\,2|S\right\rangle \right|}^{2}=0.8089\cdot 0.3968=0.3209$$

Now let us imagine that we want to analyze the path |*S*⟩→|*P*1⟩→|*E*2⟩. In this case, with respect to the previous path, the order of evidence *E2* and prediction *P1* is changed. First, we have to project the *S* state on the *P1* ray:


(34) $$\left\langle P\,1|S\right\rangle =\left[\begin{array}{cc} 10 & \end{array}\right]\,\left[\begin{array}{c} 0.2269\\ 0.9739 \end{array}\right]=\left(1\right)\cdot \left(0.2269\right)+\left(0\right)\cdot \left(0.9739\right)=0.2269$$

So the probability is:


(35) $${\left|\left\langle P\,1|S\right\rangle \right|}^{2}={\left|0.2269\right|}^{2}=0.0515$$

Then we calculate:


(36) $$\left\langle E\,2|P\,1\right\rangle =\left[\begin{array}{cc} 10 & \end{array}\right]\,\left[\begin{array}{c} 0.8994\\ 0.4372 \end{array}\right]=\left(1\right)\cdot \left(0.8994\right)+\left(0\right)\cdot \left(0.4372\right)=0.8994$$

And the probability is:


(37) $${\left|\left\langle E\,2|P\,1\right\rangle \right|}^{2}={\left|0.8994\right|}^{2}=0.8089$$

Hence the probability of the path|*S*⟩→|*P*1⟩→|*E*2⟩ is:


(38) $${\left|\left\langle E\,2|P\,1\right\rangle \right|}^{2}\cdot {\left|\left\langle P\,1|S\right\rangle \right|}^{2}=0.8089+0.0515=0.8604$$

It is important to notice that the two paths present two different probabilities, respectively, 0.3209 for the path|*S*⟩→|*E*2⟩→|*P*1⟩ and 0.8604 for the path|*S*⟩→|*P*1⟩→|*E*2⟩. This happens because:


(39) $${\left|\left\langle E\,2|S\right\rangle \right|}^{2}>{\left|\left\langle P\,1|S\right\rangle \right|}^{2}0.3968>0.0515$$

Notice also that |⟨*E*2|*P*1⟩|2=|⟨*E*1|*P*2⟩|2 because of the *law of reciprocity*. Importantly, this symmetry condition only applies to events represented by unidimensional rays, but not to such events represented by multiple dimensions (see Busemeyer and Bruza, 2012). Indeed, quantum formalism allows distinguishing between *complete measurements*—the events are identified by the rays (one-dimensional subspaces) spanned by each of the basis vectors—and *coarse measurements*—the events are not rays but whole subspaces of dimension greater than one, so that the transitions through pure or mixed states are defined. For simplicity in our stylized model, we do not consider this articulation, which is postponed to future research.

A fundamental implication of quantum formalism is the violation of the distributive axiom due to the presence of incompatible events and non-commuting projectors. The violation of the distributive axiom implies a violation of the law of total probability, which is fundamental in the Bayesian framework. Indeed, while in a Bayesian framework we know that the conditioned probability must be less than the unconditioned probability, this property is violated in the quantum framework where the probability associated with a direct path between the state vector and a final state could be less than the probability when the path is obtained through intermediate paths. For instance, let us consider the probability of the direct path |*S*⟩→|*P*1⟩ and also the mediated paths|*S*⟩→|*E*1⟩→|*P*1⟩ and |*S*⟩→|*E*2⟩→|*P*1⟩. In a quantum framework, nothing grants that the probability of the direct path is consistent with the split into two mediated paths, as happens in a Bayesian framework. Indeed, we could have the case in which the probability of the direct path |*S*⟩→|*P*1⟩ is less than the probability that considers both paths|*S*⟩→|*E*1⟩→|*P*1⟩ and |*S*⟩→|*E*2⟩→|*P*1⟩. More interestingly, we could have the case in which the probability of the direct path is also smaller than the probability of a specific mediated path. Indeed, the probability of the direct path |*S*⟩→|*P*1⟩ is |⟨*S*|*P*1⟩|2=0.0515 is smaller than the probability of the specific mediated path |*S*⟩→|*E*2⟩→|*P*1⟩, which is |⟨*P*1|*E*2⟩|2⋅|⟨*E*2|*S*⟩|2=0.3209 (see the previous sections for calculation). This property, which represents a substantial violation of the Bayesian framework and a tenet of a quantum approach, is due to the presence of *interference effects*, which (in a quantum-cognitive framework) are intended as positive or negative interferences among the intermediate states affecting the final state. In particular, when the intermediate paths are not determined, they interfere creating positive or negative interference on the probability of the final state (Busemeyer and Bruza, 2012). According to Feynman, interference is the fundamental mystery of quantum theory (Feynman et al., 1964).

The violation of the Bayesian rule, which is compatible with interference dynamics of a quantum framework (as in the well-known double-slit experiment), has been experimentally documented in the last two decades, mainly concerning judgments formulation and decision making (Busemeyer et al., 2006, 2011; Busemeyer and Bruza, 2012).

#### The Role of Quantum Contextuality

In our stylized model, incompatibility—revealed by the presence of order effects (consistently with Wang and Busemeyer, 2013)—is based on the hypothesis that top-down predictions and bottom-up evidence cannot be co-jointly determined to pursue a univocal model of (predictive) brain functioning. The nature of this “impossibility” is strictly related to the role of *quantum contextuality*. Quantum contextuality became central in quantum theory since the formulation of Bell’s well-known argument (Bell, 1966) along with its complement known as Kochen–Specker’s theorem (Kochen and Specker, 1967, see Mermin, 1993 for a retrospective overview)—discussing the Einstein–Podolsky–Rosen paradox about the incompleteness of quantum mechanics and the alleged presence of hidden variables (Einstein et al., 1935). Generally speaking, contextuality refers to the idea that measurements could be locally consistent, but they can exhibit globally inconsistency, that is to say, a random variable could change its identity depending on the conditions under which it is recorded (Liang et al., 2011; Dzhafarov and Kujala, 2014, 2015; Budroni et al., 2021). Indeed, considering a random variable as fixed under different conditions could lead to contradictions in the joint distribution with other random variables. In non-technical terms “the contextuality in a system of random variables recorded under various conditions is a deviation of the possible couplings for this system from a specifically chosen identity coupling” (Dzhafarov and Kujala, 2014, p. 8).

Discriminating the direct effects (occurring when other stimuli influence the response by changing its distribution), from “true” quantum contextuality, about such changes that cannot be explained by direct influences, is crucial in the current debate on the nature of quantum cognition. According to Dzhafarov et al. (2016), the experimental evidence related to order effects—collected by Wang and Busemeyer (2013) and explained through quantum principles—is not due to quantum contextuality, but to direct effects. Contextuality can be analyzed by eliminating all the direct influences by the experimental design, as in Cervantes and Dzhafarov (2018), experimentally discriminating the two types of effects. An operational procedure, developed specifically to compute “true” quantum contextuality in the presence of direct effects, is known as “contextuality-by-default” (Dzhafarov and Kujala, 2014, 2015). Contextuality-by-default assumes (in a Kolmogorov framework) that contextuality is normally present, that is, any two random variables under different conditions are considered different by default (for instance, instead of considering a set of fair coins as the same and unique random variable, we can identify a different random variable for each of the coins). In the special case in which they are equal with probability = 1, they are considered non-contextual.

We think that quantum contextuality, and its distinction from direct effects, represents a fundamental theoretical tool for the development of Quantum Predictive Brain (cf., Fields and Glazebrook, 2022); in particular a generalization to *N* dimensions, informed by neuro-cognitive evidence. Indeed, we can hypothesize that the interaction of different pieces of evidence can generate incompatibility whose nature should require experimentally discriminating the role of direct effects and the role of quantum contextuality. This specific development of QPB is postponed to future research.

#### The Bayesian Brain as a Special Case

When systems are compatible, as the prediction system and evidence system are orthogonal, the quantum framework becomes compatible with a classical Bayesian framework. Hence, the Bayesian brain works in the absence of complementarity between predictions and evidence, and could be considered a special case of QPB. In the case of compatibility, the classical assumptions of unicity apply, and non-commutative, order effects disappear. Indeed, when top-down processes and bottom-up processes are compatible, the assumption of unicity (fundamental in the Kolmogorov framework) is repristinated, so it is possible to form a single common sample space in which to place all the events and to define the rule of classical probability theory. A quantum framework differs from a Bayesian framework only when incompatible systems are involved (Busemeyer and Bruza, 2012).

Generally speaking, the idea that the brain is a predictive machine does not imply that it should work strictly based on Bayesian principles. Bayesian models are more familiar to most researchers, especially in the field of social and behavioral sciences. Unfortunately, such models may be limited, as they require restrictive probabilistic assumptions (for a comparison between Bayesian mechanisms and quantum probability in cognition, see Bruza et al., 2015). Using again the incipit about the deer, a Bayesian model is useful to update our model about the probability of deer crossing the road and it can be also used to improve our skills when we walk or drive in a forest. But, a Bayesian model hardly seizes the inspirational, higher-order meaning, unveiled by the metaphor and its poetical dimension. We think—and we postpone this investigation to future research—that quantum formalism offers a more flexible, generalized and sophisticated approach to predictive brain, for which the Bayesian approach represents only a specific case. Hence, QPB is not a *tout court* alternative to a Bayesian brain, but an articulation that can preserve the Bayesian updating mechanisms and can also consider them in a complementary fashion.

## Discussion

“In my childhood we were always assured that the brain was a telephone switchboard” (Searle, 1984, p. 44). The idea that new scientific ideas come from specific scientific tools is not new, as methods of statistical inference have often been translated into metaphors of mind (cf., Gigerenzer and Murray, 1987). Predictive coding is probably a case of a well-known formal device (the Bayes’ theorem was formulated long ago) used not only as an approximation of brain functioning, but also as an inspiring tool. But, if we think that this transposition of Bayesian mechanisms into the brain is only a speculative attempt, we are wrong. Predictive coding is successful not only because of its mathematical elegance, but also because of its internal consistency and explanatory power, as it proposes a successful unifying framework for many neuro-cognitive phenomena including perception, action, belief, memory and learning.

We think that what applies to the Bayesian brain applies to the QPB, which also presents the advantage of overcoming several limitations present in the Bayesian framework. Far from being just formal speculation (based on the use of a non-Kolmogorovian probability), the rationale behind a quantum approach is based on two general arguments. First, current theories of the predictive brain do not appear to be able to explain more sophisticated cognitive phenomena, involving unconventional surprises, incommensurable points of view, contemplative experience and metaphorical meaning, which are all marks of human cognition. Second, current theories of the predictive brain do not consider that predictions and evidence present an evolutionary substrate, which could be non-adaptive. Such arguments (respectively discussed in the following sections “Complementarity as a Mark of Human Cognition” and “Neural Reuse and Complementarity”) find a more natural theoretical integration in a quantum framework. A QPB offers a more flexible approach to predictive brain, able to preserve the mechanism of Bayesian updating and, at the same time, overcome its limitations through the general hypothesis of complementarity between top-down and bottom-up processes.

### Complementarity as a Mark of Human Cognition

Let us consider, again, the incipit of this contribution, “It’s not the deer that crosses the road, it’s the road that crosses the forest.” If we remain bounded in a Bayesian framework, we will consider the deer as an unexpected event and we will update our model of the forest: deer are not so much rare, so the next time we cross a forest, we should pay attention. Unfortunately, this Bayesian mechanism is as much plausible as it is trivial. The citation is not about deer living in a forest, but lies in a different cognitive order, unveiled by a metaphor (about a deer and a forest). We can conceive this metaphorical meaning only if we conceive the sensory evidence on a totally different and incompatible order of meanings. Indeed, the most interesting surprise is instantiated by the discovery of the ecological embeddedness, occurring when we realize that the road is nothing more than a small line in a huge forest. But, there is more. Once we access this higher-order meaning and we re-formulate the metaphor through an internally consistent and accurate description (the metaphor reveals our ecological embeddedness, disclosing the incapacity of conceiving our life as a part of a bigger picture), its poetical meaning suddenly disappears and becomes vague and uncertain. Notice also, when we try to express the meaning through a consistent and accurate description, new metaphors (such as the “bigger picture”) will enter again into the game of sense-making. Yet, again, there is more. There is no actual experience of a forest and a deer, our narrative is not part of a poetry book, nor are we interested in ecological embeddedness. The metaphor is a part of a scientific publication and it is instrumental to a totally different purpose and topic. Again, we found a further, higher-level order of sense-making. In short, when we are certain about a specific order of sense-making, we remain vague on the others that instantiate the sense. So, it seems to us that we can be certain about one thing precisely because of the vagueness of the complementary things.

We think that the Bayesian brain is not able to model such kinds of higher-order dynamics among different levels of metaphorical mapping, sensory evidence, poetical value, and logical consistency, and we also think that QPB presents some advantages. A fundamental implicit assumption of the Bayesian brain is that predictions and evidence are modeled as orthogonal sub-spaces. This fundamental property represents a fundamental limitation if analyzed through the quantum lens. Indeed, QPB assumes that prediction and evidence could not be independent (not orthogonal), as they can be expressed, one with respect to the other, with a precision trade-off (they are conjugate systems). The orthogonality between the prediction and evidence systems (in the Bayesian brain) is only an extreme case, and QPB explores all the other infinite possibilities of non-orthogonal but complementary interactions between prediction and evidence. Such types of incompatible interactions are central in QPB.

Speculatively, the human brain presents the “ability” of adjusting the predictive system with respect to the sensory evidence, and vice versa. Such adjustment is consistent with the well-known “adaptive resonance theory” (e.g., Grossberg, 2013), which considers the brain is organized into complementary parallel processing systems—respectively related to top-down expectations and bottom-up sensory information—whose interactions generate intelligent behaviors (Grossberg, 2000). Contrary to adaptive resonance theory—emphasizing the role of resonance among the processes—QPB considers complementarity as a phenomenon that works precisely because top-down and bottom-up processes cannot always be “reduced” one to the other, but must preserve their incompatibility to generate higher-order cognitive processes. In QPB, the adjustment between predictions and evidence—corresponding in our model to a rotation of one system with respect to the other (as shown in Figure 3)—instantiates the complementarity of the prediction, *endogenizing error minimization and precision*, both constructs being central in predictive brain theory. Furthermore, complementarity preserves the history of the dynamic interplay (through order effects) and the ability to reinvent the interaction (through interference) in a non-trivial manner. In the QPB framework, we speculatively posit that *incompatibility is necessary for higher-order surprisal*. Specific forms of surprisal (such as the deer metaphor, which makes sense precisely when one oversteps the veil of sensory obviousness) occur when the interaction between prediction and evidence presents a form of non-reducible uncertainty, requiring some indeterminism in the predictive brain (cf. De Ridder and Vanneste, 2013). Such uncertainty preserves the degrees of freedom necessary to instantiate such incommensurable-but-meaningful wild flights, which are a mark of the human being.

### Neural Reuse and Complementarity

The possibility of conceiving prediction and evidence as complementary systems is not a hypothesis that is meaningful only on a cognitive and computational level, but aims to find evidence in the areas of the brain involved in predicting and coding the sensory evidence. Despite the mounting (but also mixed) neuroimaging research on predictive coding (Walsh et al., 2020), and postulating distinct brain areas for prediction and sensory evidence, we posit that QPB is more consistent with neural reuse, hypothesizing that the same area of the brain can be used for different purposes (Gallese, 2008; Anderson, 2010; cf., Gatti et al., 2021). In particular, the possibility of conceiving the same state vector through incompatible descriptions is a way of modeling neural reuse, where the same neural endowments admit a representation both in the predictive system and in the evidence system. Hence, the possibility of adjusting the prediction and the sensory-motor systems, preserving their incompatibility, is strictly related to the idea of exploiting the same neural resources for both predicting and coding the sensory evidence. Incompatibility is what preserves the specificities of prediction and evidence systems within the same neural resources; speculatively, when a brain area is involved in prediction, its role in coding evidence is uncertain, and vice versa, that is to say, we hypothesize that the same neural endowment enables partial determinations in the form of prediction or evidence.

In QPB, predictions and evidence are not necessarily distinct brain areas, but they are analytical distinctions that identify specific functions, which share some neural resources. This fundamental mechanism—the same neural resources for different functions—challenges a fundamental property assumed in existing predictive brain theories, that is, the existence of a strictly hierarchical organization of the brain. QPB proposes an alternative topological model that shifts the theoretical focus *from layers to order*s. If we admit that the same neural resources enable different functions, we are substituting the necessity for layers with the possibility of instantiating specific functions, without a strict functional topology but through complementarity. Indeed, predictions and evidence can be determined only by assuming that the same neural resources collapse in a system, while preserving the indeterminacy of the complementary system in a dynamic, non-commutative fashion. It can be noticed, again speculatively, that by avoiding a strictly hierarchical organization and allowing neural “shortcuts”, complementarity can be also useful to shed new light on neuroplasticity. More specifically, a Bayesian brain is not able to fully account for such predictions that, being phylogenetically-determined adaptations, are resistant to contextual changes (Yon et al., 2019). A QPB, being consistent with neural reuse, preserves both the adaptive functions and the degree of freedom for the instantiation of high-level processes, to be intended as *exaptations* of the sensory-motor system (Anderson, 2007; Mastrogiorgio et al., 2022).

Hence, we do not deny that the human brain presents hierarchies in the topology of neural networks. However, we think that the strict hierarchical organization assumed in current theories of predictive brain seems to be more related to the necessity of formulating a theory consistent with the Bayesian framework, than to the opportunity of interpreting the flourishing neuroimaging evidence through the Bayesian lens. Put differently, it seems to us that a Bayesian framework imposes specific and restrictive topological conditions to preserve its theoretical consistency. This limitation is one that QPB tries to overcome, endorsing the view that specific old brain structures are, evolutionarily speaking, exapted to enable high-level faculties employed in civilized niches.

## Conclusion

While quantum cognition is, nowadays, mostly applied to high-level processes, mainly in a computationalist, cognitivist framework, QPB exploits quantum formalism to shed new light on the role of embodied mechanisms in a predictive brain framework. QPB is not a representationalist theory to be ascribed to a pure “cognitive realm,” in which quantum principles have been applied to model high-level information processing (e.g., Khrennikov, 2006, 2015; Aerts, 2009; Asano et al., 2015), nor is it consistent with the controversial hypothesis—also known as Orchestrated objective reduction (Orch OR)—stating that consciousness originates from quantum states in microtubules (Hameroff, 2012; Hameroff and Penrose, 2014).

QPB assumes that the brain is a predictive machine working through conservative principles and proposes a quantum model that is able to seize the complementarity between top-down and bottom-up processes, within a predictive coding framework. QPB challenges the implicit assumption of commensurability between predictions and evidence (assumed in current views of predictive brain) postulating that incompatibility is a mark of human cognition. QPB assumes that prediction and evidence could not be independent but complementary, as they can be determined, one with respect to the other, only with a precision trade-off. Complementarity—to be considered necessary for higher-order surprisal—preserves the history of the dynamic interplay of the systems (through order effects) and their non-trivial interaction (through interference). QPB is an evolutionary friendly framework, consistent with neural reuse (as the same neural endowment enables partial descriptions in the form of prediction or evidence) and can shed new light on neuroplasticity.

While predictive coding, based on Bayesian principles, is a consolidated domain of research, we are aware that QPB is a novel and speculative hypothesis. But, we think that our hypothesis is no less speculative than a Bayesian brain, as a Bayesian approximation can be considered a special case of QPB. We also think that QPB presents theoretical parsimony and elegance, as it allows considering the updating mechanisms also in a complementary fashion, endogenizing precision and error minimization (without requiring *ad hoc* assumptions).

Generally speaking, predictive brain (on the one side) and quantum cognition (on the other) are two different domains of investigation, each one characterized by well-developed theoretical debates connoted by advanced, dedicated mathematical formalism. We prudently opted for a stylized—and speculative—model, precisely because of the scientific inopportunity of matching the theoretical complexity of such distinct domains of research (this type of matching has been proposed by Fields et al., 2021). Notice that our stylized quantum model is deliberately paired with a stylized representation of a Bayesian brain. A less-stylized, more advanced quantum model that is able to take into account more recent articulation of research on active inference is postponed to future research.

In conclusion, we think that QPB could represent a promising framework for future investigations on the predictive nature of the human brain. We also think that QPB deserves the deepest theoretical articulation along with experimental research and neuroimaging evidence to be fully substantiated. Building a realistic model of the QPB represents a challenge for future research. Such a model should be characterized by N dimensions specified on the basis of neuro-cognitive evidence and should take into account the role of quantum contextuality.

## Data Availability Statement

The original contributions presented in this study are included in the article/supplementary material, further inquiries can be directed to the corresponding author.

## Author Contributions

The author confirms being the sole contributor of this work and has approved it for publication.

## Conflict of Interest

The author declares that the research was conducted in the absence of any commercial or financial relationships that could be construed as a potential conflict of interest.

## Publisher’s Note

All claims expressed in this article are solely those of the authors and do not necessarily represent those of their affiliated organizations, or those of the publisher, the editors and the reviewers. Any product that may be evaluated in this article, or claim that may be made by its manufacturer, is not guaranteed or endorsed by the publisher.
